# METTL3 aggravates lung injury in neonatal mice with *Streptococcus pneumoniae*-induced pneumonia via the circ_0001239/KLF10 axis

**DOI:** 10.1128/iai.00288-25

**Published:** 2025-10-17

**Authors:** Liping Yang, Yufei Xie, Panpan Yan, Mei Liu, Jingjing Zhang, Caixia Ma

**Affiliations:** 1 Department of Pediatrics, Qingpu Branch of Zhongshan Hospital, Fudan University 668070, Shanghai, China; University of Illinois Chicago, Chicago, Illinois, USA

**Keywords:** METTL3, pneumonia, *Streptococcus pneumoniae*, inflammation, lung injury

## Abstract

As a leading causative agent of pneumonia infection worldwide, *Streptococcus pneumoniae* (*Spn*) induces lung injury and presents substantial therapeutic challenges. To elucidate the role of methyltransferase-like 3 (METTL3) in modulating circular RNA_0001239 (circ_0001239), YTH domain containing protein 2 (YTHDC2), and Krüppel-like factor 10 (KLF10) through m6A modification, we established *Spn*-induced neonatal mouse models. The survival rates, bacterial load in bronchoalveolar lavage fluid, and METTL3 expression in pulmonary tissue were evaluated. After METTL3 downregulation, lung wet-to-dry ratio, myeloperoxidase activity, and inflammatory markers were assessed. Methylated RNA immunoprecipitation detected enriched m6A modification on circ_0001239, while RNA immunoprecipitation validated the bindings of circ_0001239 to YTHDC2 and YTHDC2 to KLF10. The KLF10 mRNA stability was analyzed via actinomycin D treatment. METTL3 and circ_0001239 were upregulated in pneumonic lungs, while KLF10 was downregulated. METTL3 knockdown improved survival, alleviated lung injury, increased superoxide dismutase levels, and suppressed interleukin (IL)-6, IL-1β, and malondialdehyde levels. METTL3 promoted the binding of circ_0001239 to YTHDC2 via m6A modification, destabilizing KLF10 mRNA. Circ_0001239 overexpression or KLF10 knockdown reversed the protective effects of low expression of METTL3 on lung damage in neonatal mice with pneumonia. In conclusion, METTL3 aggravates *Spn*-induced lung injury via m6A-dependent circ_0001239/YTHDC2/KLF10 axis, thereby providing potential therapeutic targets for severe pneumonia.

## INTRODUCTION

Pneumonia is a leading cause of high morbidity that severely impacts respiratory health through alveolar and airway damage (1). As an important cause and complication of lung injury, pneumonia exhibits a bidirectional relationship between its pathological progression and lung injury (2). Pneumonia can be caused by various etiologic agents, including bacteria, viruses, and atypical organisms (3). *Streptococcus pneumoniae* (*Spn*) is a gram-positive bacterium that can lead to several diseases, such as pneumonia, bacteremia, and meningitis (4). Therefore, the primary objective of this study is to elucidate the roles of pneumonia-related molecules in *Spn*-induced pneumonia and their contribution to lung injury progression while validating the promotion effect of pneumonia-targeted therapies on pulmonary tissue repair in neonatal mouse models.

N6-methyladenosine (m6A) represents the most common, abundant, and conserved internal modification occurring co-transcriptionally in eukaryotic RNAs, playing critical roles in the development and progression of numerous human cancers (5). Methyltransferase-like 3 (METTL3), as the most extensively discussed m6A methyltransferase, regulates post-transcriptional gene expression and is involved in cellular proliferation, differentiation, and inflammatory responses (6, 7). METTL3 drives the progression of lung injury by regulating m6A-dependent pro-inflammatory gene expression and represents a potential therapeutic target (8). In neonatal mouse and cell models of bacterial pneumonia, METTL3 expression is upregulated in association with lung injury and positively correlates with the severity of pulmonary inflammation (9–11). METTL3 exacerbates *Spn*-induced lung injury through enhancing alveolar epithelial cell apoptosis and inflammatory responses (12). However, the specific downstream pathway of METTL3 in *Spn*-triggered pneumonia remains to be elucidated. Therefore, this study investigated the effect of METTL3 on the expression of key inflammatory mediators during *Spn* infection via m6A modification.

Circular RNAs (circRNAs) are covalently closed single-stranded RNAs widely present in eukaryotes (13). CircRNAs play critical roles in the pathogenesis, progression, and therapeutic management of lung injury through regulating gene transcriptional activity, controlling protein translation, and binding to various protein components and microRNAs (miRNAs) to modulate their function while also serving as potential biomarkers and therapeutic targets (14). For instance, circ_0001239 enhances LPS-induced pulmonary cell apoptosis, inflammatory response, and oxidative stress, ultimately exacerbating lung injury in pneumonia (15). Studies indicate that m6A-modified circRNAs participate in the development of inflammation (16–18). Therefore, we inferred that METTL3 might upregulate circ_0001239 expression through m6A modification to aggravate lung inflammatory injury.

KLF10 (Krüppel-like factor 10), a member of the Krüppel-like factor family, is known to regulate inflammatory responses and tissue repair (19). Loss of KLF10 results in increased lung inflammation (20). Building on our research findings, we innovatively propose a hypothesis that METTL3 dynamically regulates circ_0001239 through m6A modification and drives the recruitment of YTH domain containing protein 2 (YTHDC2) to circ_0001239, thereby regulating KLF10 expression to aggravate *Spn*-induced inflammatory cascades, which may offer a new direction for treating pneumonia-associated lung injury.

This research seeks to elucidate the regulatory mechanism of METTL3 in lung injury in neonatal mice with *Spn*-induced pneumonia, establishing novel theoretical foundations for therapeutic strategies for pneumonia.

## MATERIALS AND METHODS

### Animal experiments

Male BALB/c mice (3 weeks old, 12–15 g) purchased from Vital River Laboratory Animal Technology (Beijing, China) were housed under controlled conditions with a temperature of 20–25°C and a 12 h light/dark cycle (12 h illumination, followed by 12 h darkness). Mice had free access to food and water throughout the study.

### Preparation of *Spn* and lentivirus

*Spn* (serotype 19F [Danish], type 19 [U.S.], ATCC49619, American Type Culture Collection, Rockville, MD, USA) was inoculated onto Todd-Hewitt yeast extract (THY) sheep blood agar (Shanghai Aiyan Biotechnology Co., Ltd., Shanghai, China) and incubated at 37°C in a 5% CO_2_ atmosphere for 18 h. Bacteria were harvested and subsequently re-suspended in sterile phosphate-buffered saline (PBS, 0.15 M, pH 7.2) to 10^9^ colony-forming units (CFU)/mL. Lentiviruses containing METTL3 shRNA (sh-METTL3), circ_0001239 overexpression vector (circ), YTHDC2 shRNA (sh-YTHDC2), KLF10 shRNA (sh-KLF10), and their corresponding negative controls (sh-NC, NC) were obtained from Gemma (Shanghai, China).

### Establishment of an *Spn*-infected neonatal pneumonia mice model and lentivirus injection

According to the previous report (21), the *Spn*-induced pneumonia model was established in neonatal mice. In brief, anesthesia was induced in mice via intraperitoneal administration of sodium pentobarbital at a dose of 50 mg/kg (Sigma, St. Louis, MO, USA), followed by bilateral intranasal inoculation with 1 × 10^8^ CFU *Spn* suspended in PBS (50 µL per nostril) to establish a pneumonia model (*Spn* group). The mice in the sham group were injected with sterile PBS as a control. Lentivirus was administered into mice via tail vein injection (the viral titer was 1 × 10^9^ TU/mL, and the volume was 3 µL), followed by induction of the pneumonia model 2 days later (22). Survival rates were monitored daily for 14 days. At the experimental endpoint, mice were sacrificed via intraperitoneal administration of 100 mg/kg pentobarbital sodium. Each group was randomly assigned for: lung wet-to-dry weight ratio analysis (*N* = 6), bronchoalveolar lavage fluid (BALF) collection (*N* = 6), hematoxylin and eosin (H&E) staining, and tissue homogenate assays (*N* = 6).

### Bacterial load in BALF

The collected BALF was centrifuged for 10 min, and the resulting pellet was resuspended in sterile PBS at a volume of 0.5 mL. Serial 10-fold dilutions of the suspension were performed, with 50 mL of the final dilution plated onto sheep blood agar. The culture plates were maintained under 5% CO₂ condition at 37°C for an 18 h incubation period, after which CFUs were quantified.

### Lung wet-to-dry weight ratio

The wet weight of fresh pulmonary tissue was calculated using an electronic balance and recorded. Subsequently, the tissue was processed in an oven at 70°C until a constant weight (dry weight) was achieved, and the dry weight was documented. Wet-to-dry weight ratio = wet weight/dry weight.

### Myeloperoxidase (MPO) activity

The collected pulmonary tissue was homogenized in Hank’s balanced salt solution and centrifuged for 20 min, and then the supernatant was collected. The MPO activity in the lung homogenates was determined using a kit (Colorimetric; ab105136, Abcam, Cambridge, MA, USA) in strict accordance with the manufacturer’s protocol.

### H&E staining

The collected pulmonary tissue was fixed in 4% paraformaldehyde for 24 h, followed by graded ethanol dehydration and paraffin embedding. Sections were dried, dewaxed, stained with hematoxylin (Solarbio, Beijing, China) for 5 min, counterstained with 5% eosin solution (Solarbio) for 3 min, then dehydrated, cleared, and mounted for the morphological observation of the pulmonary tissue under light microscopy (Olympus, Tokyo, Japan) (22). Pathological scores were assessed by pathologists using a double-blind approach according to the 0–3 scoring system (0 = normal; 1 = mild; 2 = moderate; 3 = severe for interstitial/alveolar edema, hemorrhage, alveolar septal thickening, and infiltration of inflammatory cells) (23). In some of the H&E images, some marks or artifacts may be present in some histology images due to blade scratches from the sectioning process. These scratches can result in variations in tissue thickness across different regions, leading to subsequent differences in light exposure during imaging. Such imperfections arise from technical challenges in experimental procedures.

### Measurement of IL-6, IL-1β, superoxide dismutase (SOD), and malondialdehyde (MDA)

The levels of interleukin (IL)-6 (ab222503, Abcam) and IL-1β (ab197742, Abcam) in pulmonary tissue supernatants were measured using enzyme-linked immunosorbent assay (ELISA) kits following the manufacturer’s instructions, with absorbance determined by a microplate reader (Bio-Rad, Hercules, CA, USA). Superoxide dismutase (SOD) and malondialdehyde (MDA) levels in supernatants were assessed using SOD (S0101M, Beyotime, Shanghai, China) and MDA assay kits (S0131M, Beyotime) according to the manufacturer’s instructions.

### Culture and treatment of lung epithelial cells

The murine lung epithelial cells (MLE-12) were purchased from the Shanghai Institute of Biochemistry and Cell Biology (Shanghai, China) and maintained in DMEM/F-12 medium supplemented with 100 µg/mL streptomycin, 100 U/mL penicillin, and 10% fetal bovine serum cultured at 37°C in a humidified 5% CO_₂_ atmosphere. The cells were transduced with lentivirus at a multiplicity of infection (MOI) of 20, and stable cell lines were selected for subsequent experiments.

### N6-methyladenosine (m6A) quantification analysis

The relative abundance of m6A in total RNA was measured using the EpiQuik m6A RNA methylation quantification kits (Colorimetric method) (P-9005, Epigentek, Farmingdale, NY, USA) following the manufacturer’s explanations. Briefly, 200 ng RNA was incubated with a solution containing anti-m6A antibodies. Absorbance values at 450 nm were utilized in the colorimetric quantification of m6A modification levels.

### Methylated RNA immunoprecipitation (MeRIP) and RNA immunoprecipitation (RIP)

The anti-m6A antibody (ab151230, Abcam), anti-YTHDC2 antibody (ab220160, Abcam), and anti-IgG antibody (ab172730, Abcam) were conjugated to protein A/G magnetic beads within the IP buffer solution (140 mM NaCl, 1% NP-40, 2 mM EDTA, 20 mM Tris pH 7.5) overnight at 4°C. Total RNA was incubated with the antibody in IP buffer, followed by elution of precipitated RNA from the beads. At last, the eluted RNA was reverse-transcribed and analyzed by real-time quantitative polymerase chain reaction (RT-qPCR).

### Actinomycin D (Act D) treatment

Cells were exposed to Act D (Sigma) and collected at 0, 4, 6, and 8 h post-treatment. Total RNA was isolated from the treated cells and subjected to RT-qPCR analysis.

### Database analysis

The Starbase database (http://starbase.sysu.edu.cn/index.php) (24) was used to predict the binding relationships of circ_0001239, YTHDC2, and KLF10. The SRAMP database (http://www.cuilab.cn/m6asiteapp/old) (25) was used to predict m6A modifications on circ_0001239.

### RT-qPCR

Total RNA extraction from tissue samples or cultured cells was conducted with Trizol reagent (Invitrogen, Carlsbad, CA, USA) according to the manufacturer’s protocol. Reverse transcription was carried out using the Prime Script RT Kit (Takara, Dalian, China). Real-time PCR analysis was conducted on ABI 7500 Real-Time PCR System (Applied Biosystems, Foster City, CA, USA) using SYBR Premix Ex Taq II (Takara). Glyceraldehyde-3-phosphate dehydrogenase (GAPDH) served as an internal reference gene, and the relative expression levels were calculated using the 2^−ΔΔCt^ method (26). The primer sequences are shown in Table 1.

**TABLE 1:** PCR primer sequences

Gene	Sequences (5′−3′)
METTL3	F: TCTTGCCATCTCTACGCCAG
	R: GGAGGAGACCTCGCTTTACC
circ_0001239	F: GCTGATTCTGATGACAGTGCT
	R: TGACTCTTTCCAATCTGACGC
KLF10	F: GCTCGCTCCGATGAACTGTC
	R: GCGAGATGCTGAGGGCAAGA
YTHDC2	F: TGACCAGTACGGAAAGAGCC
	R: GGTCATCATTGCATGAGCTGT
GAPDH	F: GGTCCCAGCTTAGGTTCATCA
	R: AATCCGTTCACACCGACCTT

### Western blot

Cells were washed twice with ice-cold PBS and lysed in radioimmunoprecipitation assay (RIPA) lysis buffer (CW Biotech, Beijing, China) supplemented with protease inhibitors (Roche Diagnostics, Basel, Switzerland). The pulmonary tissues were homogenized using RIPA lysis buffer (Beyotime) containing 1% phenyl methane sulfonyl fluoride (Beyotime), and the protein concentration was determined using a bicinchoninic acid protein assay kit (Thermo Fisher Scientific, Waltham, MA, USA). An equivalent quantity of proteins was resolved using 10% sodium dodecyl sulfate polyacrylamide gel electrophoresis (SDS-PAGE) and subsequently transferred onto polyvinylidene fluoride membranes. Membranes were blocked with 5% skim milk at room temperature for 2 h, and then incubated with the following primary antibodies: METTL3 (1:1,000, ab195352, Abcam), KLF10 (1:1,000, ab73537, Abcam), YTHDC2 (1:1,000, ab220160, Abcam), and β-actin (1:1,000, ab8227, Abcam). Membranes were subsequently incubated with secondary antibodies (1:2,000, ab205718, Abcam) for 1 h at room temperature. β-Actin served as the endogenous control. Protein bands were visualized using the ECL Plus Chemiluminescence Kit (Pierce, Rockford, IL, USA) and captured with a chemiluminescence imaging system. Band intensity quantification was conducted using ImageJ software. In some of the western blot images, some artifact lines may be present and are likely caused by scratches on the original film during the experiment.

### Statistical method

All statistical analyses and graphical representations were performed using SPSS 21.0 (IBM SPSS Statistics, Chicago, IL, USA) and GraphPad Prism 8.0 (GraphPad Software Inc., San Diego, CA, USA). First, normality and homogeneity of variance were verified through statistical testing, confirming both a normal distribution pattern and equality of variances across groups. Comparisons among multiple groups were evaluated using one-way or two-way analysis of variance (ANOVA), followed by Tukey’s multiple comparisons test. *P* < 0.05 indicated statistical significance.

## RESULTS

### METTL3 is upregulated in neonatal mice with *Spn*-induced pneumonia and associated with lung injury

**Fig 1:**
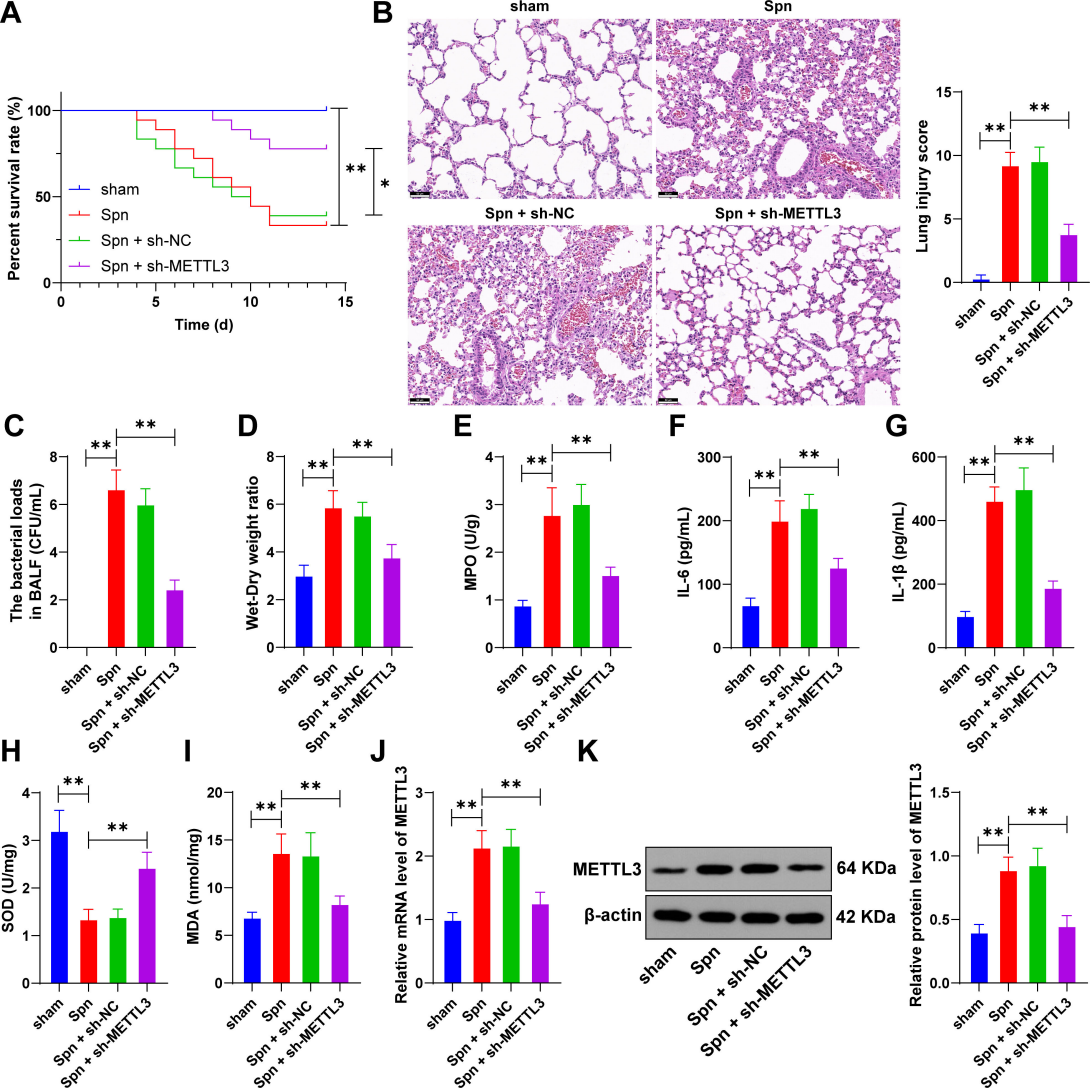
METTL3 is upregulated in neonatal mice with *Spn*-induced pneumonia and associated with lung injury. Neonatal mice were injected via tail vein with lentivirus containing METTL3 shRNA (sh-METTL3) using lentivirus with NC shRNA (sh-NC) as the negative control. Subsequently, a pneumonia model in neonatal mice was established through *Spn* induction. (**A**) Recording the survival rate of neonatal mice within 14 days; (**B**) H&E staining and damage score of the lung tissue; (**C**) bacterial load in BALF; (**D**) wet-to-dry weight ratio in lung tissue; (**E**) MPO activity in the lung tissue; (**F**) the levels of IL-6 in the lung tissue were detected by ELISA; (**G**) the levels of IL-1β in the lung tissue were detected by ELISA; (**H**) the levels of SOD in the lung tissues were detected by kit; (**I**) the levels of MDA in the lung tissues were detected by the kit; and (**J, K**) the expression of METTL3 in the lung tissue was detected using RT-qPCR and western blot. Animal experiments were conducted with *N* = 6, and data are presented as mean ± SD. Comparisons among multiple groups in (**A**) were detected using log-rank; comparisons among multiple groups in (**B–K**) were performed using one-way ANOVA, followed by Tukey’s multiple comparisons test. * *P* < 0.05. ** *P* < 0.01.

The role of METTL3 in lung injury of neonatal mice with *Spn*-induced pneumonia remains unclear. Thus, we established an *Spn*-induced pneumonia model in neonatal mice. Compared with the sham group, the *Spn* group exhibited decreased survival rates, aggravated pulmonary tissue damage, and elevated bacterial load in BALF (*P* < 0.01, Fig. 1A through C). The *Spn* infection significantly increased lung wet-to-dry weight ratios and MPO activity (*P* < 0.01, Fig. 1D and E). Moreover, *Spn* treatment significantly increased the concentrations of IL-1β, IL-6, and MDA and reduced SOD activity (*P* < 0.01, Fig. 1F through I). RT-qPCR and western blot showed that METTL3 expression was upregulated in the pulmonary tissues of neonatal mice in the *Spn* group (*P* < 0.01, Fig. 1J and K). Next, after METTL3 expression was downregulated via lentivirus injection (*P* < 0.01, Fig. 1J and K), survival rates were increased; pulmonary tissue injury was reduced; and bacterial load in BALF was decreased (*P* < 0.05, Fig. 1A through C). METTL3 inhibition decreased lung wet-to-dry ratios and MPO activity, reduced the levels of IL-1β, IL-6, and MDA, and increased the SOD activity (*P* < 0.01, Fig. 1D through I). These results demonstrate that METTL3 upregulation correlates with lung injury in neonatal mice with *Spn*-induced pneumonia.

### METTL3 upregulates circ_0001239 expression through m6A modification

**Fig 2:**
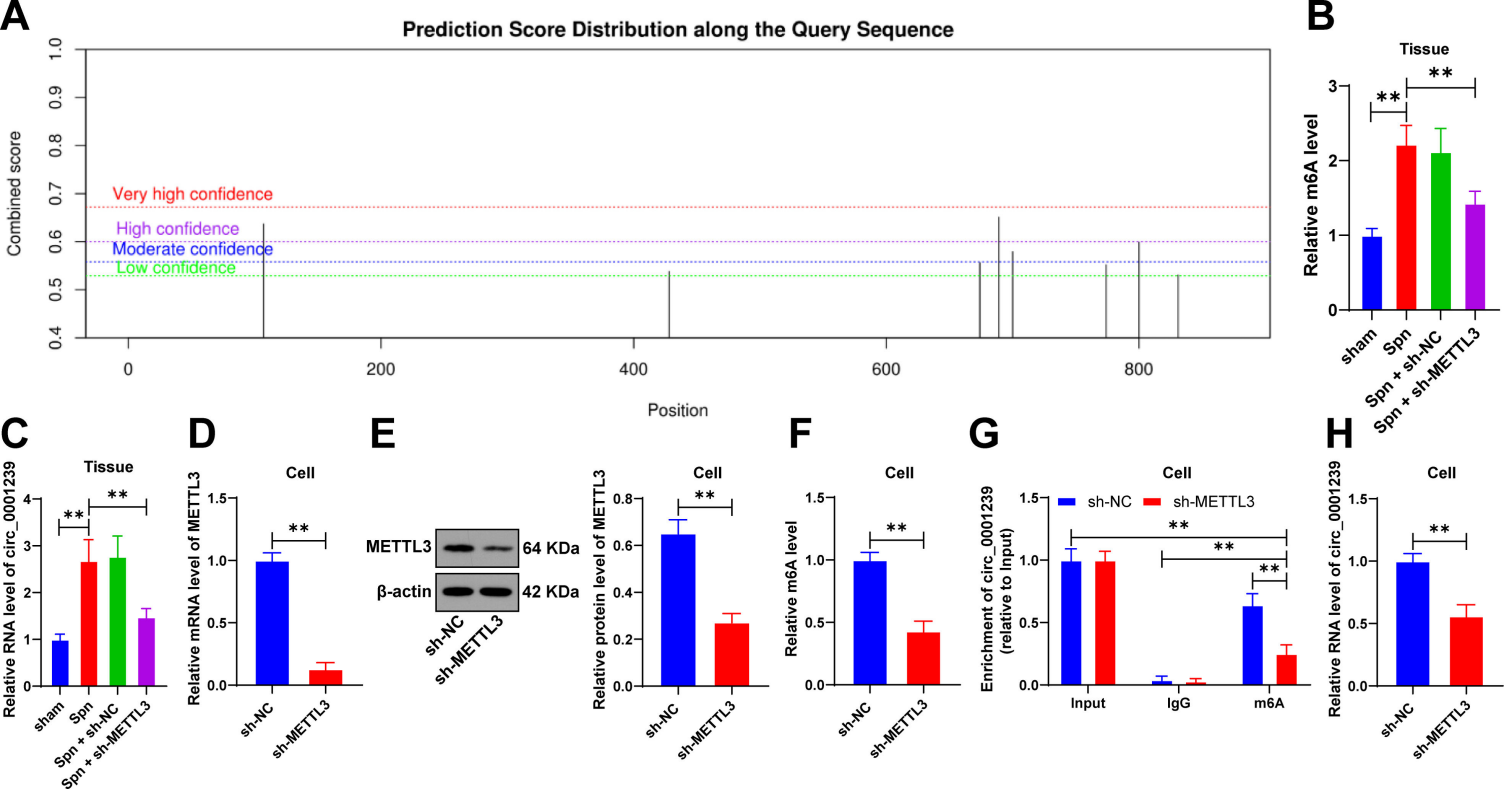
METTL3 upregulates the circ_0001239 expression through m6A modification. (**A**) m6A modification sites of circular RNA circ_0001239 predicted by the SRAMP database; (**B**) m6A levels in lung tissues were measured using the EpiQuik m6A RNA Methylation Quantification Kit; (**C**) Circ_0001239 expression in lung tissues was detected via RT-qPCR; murine lung epithelial cells (MLE-12) were cultured and transfected with sh-METTL3 using sh-NC as a negative control; (**D, E**) METTL3 transfection efficiency in cells was validated by RT-qPCR and western blot; (**F**) m6A levels in cells were determined using the EpiQuik m6A RNA Methylation Quantification Kit; (**G**) m6A enrichment on circ_0001239 in cells was evaluated using the MeRIP assay; (**H**) the expression of circ_0001239 in cells was detected by RT-qPCR. Animal experiments were conducted with *N* = 6, and cell experiments with *N* = 3. Data are presented as mean ± SD. Comparisons between two groups in (**D–F and H**) were performed using *t*-test; comparisons among multiple groups in (**B, C**) were performed using one-way ANOVA, while in (**G**) were analyzed by two-way ANOVA, followed by Tukey’s multiple comparisons test. ** *P* < 0.01.

Database prediction indicated that circ_0001239 contained m6A modification sites (Fig. 2A), and the circ_0001239 expression is increased in lung injury (15). However, the regulatory role of METTL3 on circ_0001239 remains unclear. First, based on the detection results of the EpiQuik m6A RNA Methylation Quantification Kit in lung tissues, we observed elevated m6A levels in the *Spn* group compared to the sham group, whereas m6A levels were reduced in the *Spn* + sh-METTL3 group compared to the *Spn* + sh-NC group (*P* < 0.01, Fig. 2B). Next, we measured the circ_0001239 expression and found that the circ_0001239 expression was upregulated in the pulmonary tissue of neonatal mice with *Spn*-induced pneumonia but was downregulated in the pulmonary tissue with low expression of METTL3 (*P* < 0.01, Fig. 2C). To validate the mechanism in cell models, MLE-12 was transfected with sh-METTL3, revealing that intracellular m6A levels were decreased after METTL3 downregulation (*P* < 0.01, Fig. 2D through F). Following METTL3 downregulation, m6A enrichment on circ_0001239 was diminished (*P* < 0.01, Fig. 2G), and circ_0001239 expression in cells was reduced (*P* < 0.01, Fig. 2H). Collectively, METTL3 upregulates circ_0001239 expression via m6A modification.

### Circ_0001239 overexpression attenuates the protective effects of METTL3 knockdown on neonatal mice with *Spn*-induced pneumonia

**Fig 3:**
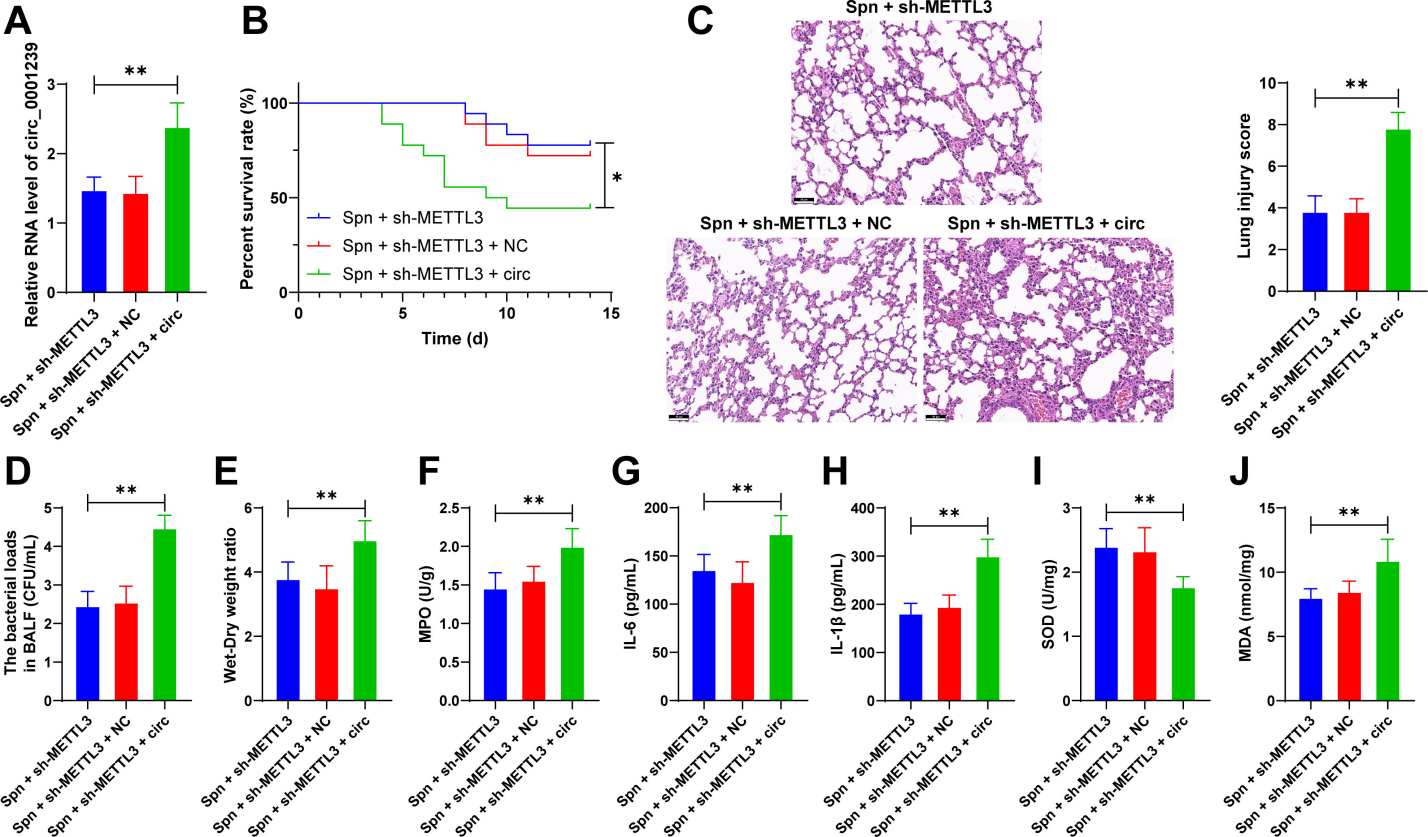
Circ_0001239 overexpression attenuates the protective effects of METTL3 knockdown on neonatal mice with *Spn*-induced pneumonia. Neonatal mice were injected via the tail vein with lentivirus containing overexpressing circ_0001239 vectors (circ group), while those receiving empty vector (NC group) served as the negative control. Subsequently, the model of neonatal mice with *Spn*-induced pneumonia was established. (**A**) Circ_0001239 expression in lung tissues was detected using RT-qPCR analysis; (**B**) survival rate of neonatal mice monitoring within 14 days; (**C**) H&E staining and damage score of lung tissue; (**D**) bacterial load in BALF; (**E**) lung wet-to-dry weight ratio measurement; (**F**) MPO activity assessment in the lung tissue; (**G**) IL-6 levels in the lung tissue were determined by ELISA; (**H**) IL-1β levels in the lung tissue were determined by ELISA; (**I**) SOD concentrations were quantified using assay kits; and (**J**) MDA concentrations were quantified using assay kit. All animal experiments were performed with *N* = 6, with data expressed as mean ± SD. Comparisons among multiple groups in (**B**) were performed using log-rank test; comparisons among multiple groups in (**A and C–J**) were analyzed by one-way ANOVA, followed by Tukey’s multiple comparisons test. * *P* < 0.05. ** *P* < 0.01.

Subsequently, we upregulated circ_0001239 expression in pulmonary tissues of neonatal mice through lentiviral injection (*P* < 0.01, Fig. 3A), which resulted in reduced survival rates, aggravated pulmonary tissue injury, and increased bacterial load in BALF (*P* < 0.05, Fig. 3B through D). Furthermore, circ_0001239 overexpression led to elevated lung wet-to-dry weight ratios, MPO activity, IL-1β, IL-6 and MDA levels, and decreased SOD activity (*P* < 0.01, Fig. 3E through J). In conclusion, these findings suggest that circ_0001239 overexpression attenuates the protective effects of METTL3 downregulation on *Spn*-induced pneumonia in neonatal mice.

### The interaction between circ_0001239 and the RNA-binding protein YTHDC2 leads to KLF10 downregulation

**Fig 4:**
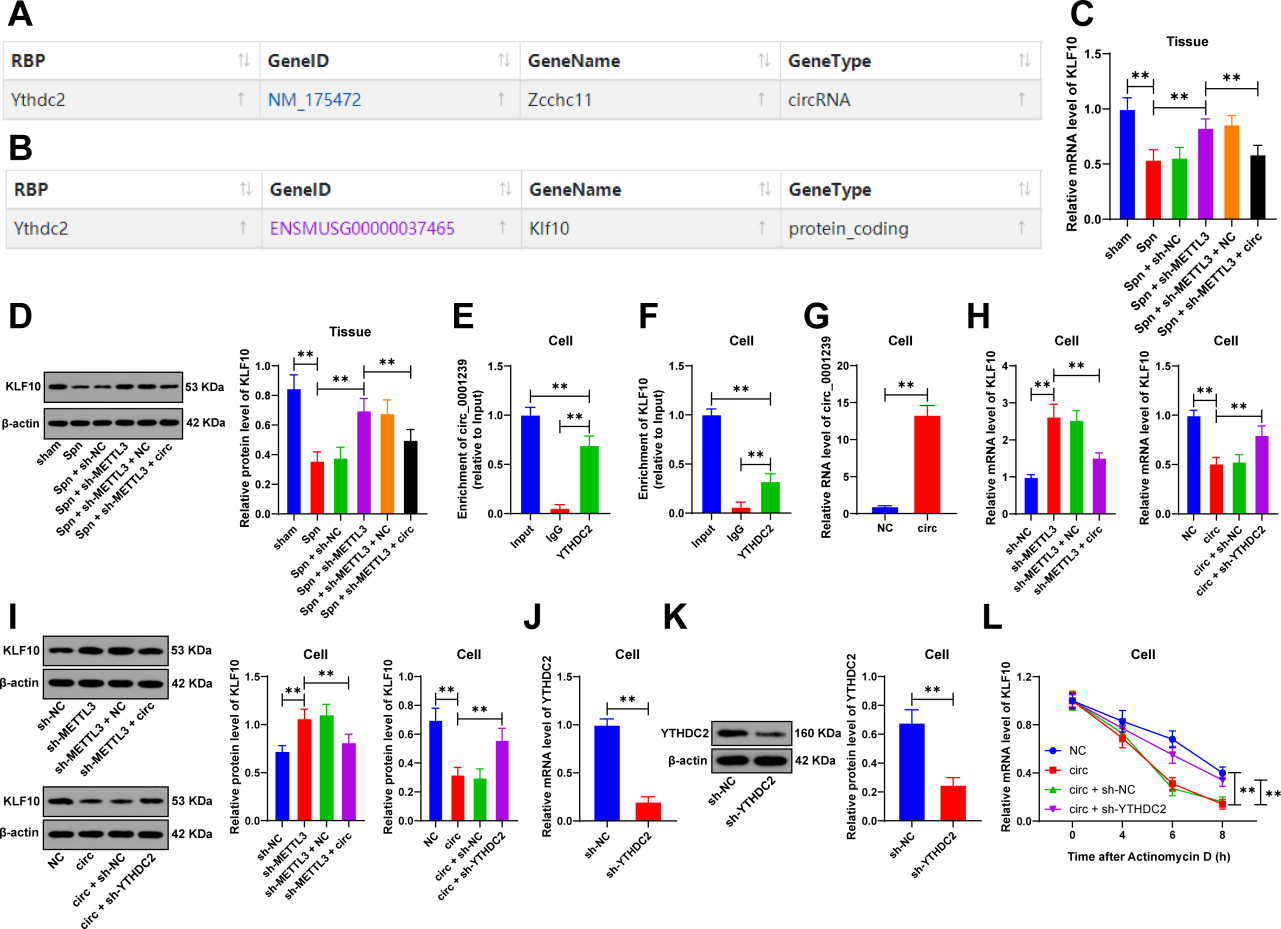
The interaction between circ_0001239 and the RNA-binding protein YTHDC2 leads to KLF10 downregulation. (**A**) Starbase prediction of interactions between circ_0001239 and the RNA-binding protein YTHDC2; (**B**) Starbase prediction of interactions between the RNA-binding protein YTHDC2 and KLF10; (**C, D**) KLF10 expression in lung tissues was detected by RT-qPCR and western blot; murine lung epithelial cells (MLE-12) were cultured and infected with lentivirus; (**E**) binding relationships between circ_0001239 and YTHDC2 in cells were analyzed by RIP assay; (**F**) binding relationships between YTHDC2 and KLF10 in cells were analyzed by RIP assay; (**G**) infection efficiency of circ_0001239 in cells was measured by RT-qPCR; (**H, I**) KLF10 expression in cells was assessed by RT-qPCR and western blot; (**J, K**) infection efficiency of YTHDC2 in cells was evaluated by RT-qPCR and western blot; and (**L**) mRNA stability of KLF10 in cells was determined by RT-qPCR. All animal experiments were independently repeated six times, and cellular experiments were repeated three times, with data presented as mean ± SD. Comparisons between two groups in (**G and J, K**) were performed using *t*-test; Comparisons among multiple groups in (**C–F**) and (**H, I**) were analyzed by one-way ANOVA, and in (**L**) were analyzed by two-way ANOVA, followed by Tukey’s multiple comparisons test. ** *P* < 0.01.

The interaction between circRNA and RNA-binding proteins results in the downregulation of downstream gene expression (27). The Starbase database analysis predicted RNA-binding proteins potentially interacting with circ_0001239. Among the candidates, YTHDC2 demonstrated the highest predicted binding score (Fig. 4A). YTHDC2 was selected as the RNA-binding protein interacting with circ_0001239 in this study. Subsequently, we utilized the Starbase database to predict factors capable of binding to YTHDC2, and KLF10 emerged as a notable candidate (Fig. 4B). The deficiency of KLF10 had been shown to exacerbate pulmonary inflammatory responses (20). Experimental data showed that KLF10 expression was decreased following *Spn* treatment in lung tissues, increased upon METTL3 downregulation, and decreased again after circ_0001239 overexpression (*P* < 0.01, Fig. 4C and D). In cell models, RIP assay showed that compared with IgG, YTHDC2 could be enriched on circ_0001239, and YTHDC2 could also be enriched on KLF10 (*P* < 0.01, Fig. 4E and F). METTL3 downregulation elevated KLF10 expression, while circ_0001239 upregulation (*P* < 0.01, Fig. 4G) suppressed KLF10 expression (*P* < 0.01, Fig. 4H and I). Furthermore, circ_0001239 overexpression decreased KLF10 mRNA stability, whereas YTHDC2 knockdown (*P* < 0.01, Fig. 4J and K) enhanced KLF10 mRNA stability (*P* < 0.01, Fig. 4L), with corresponding changes observed in KLF10 protein levels in cells (*P* < 0.01, Fig. 4H and I). To sum up, circ_0001239 interacts with YTHDC2, leading to downregulation of KLF10.

### KLF10 downregulation alleviates the protective effect of METTL3 knockdown in neonatal mice with *Spn*-induced pneumonia

**Fig 5:**
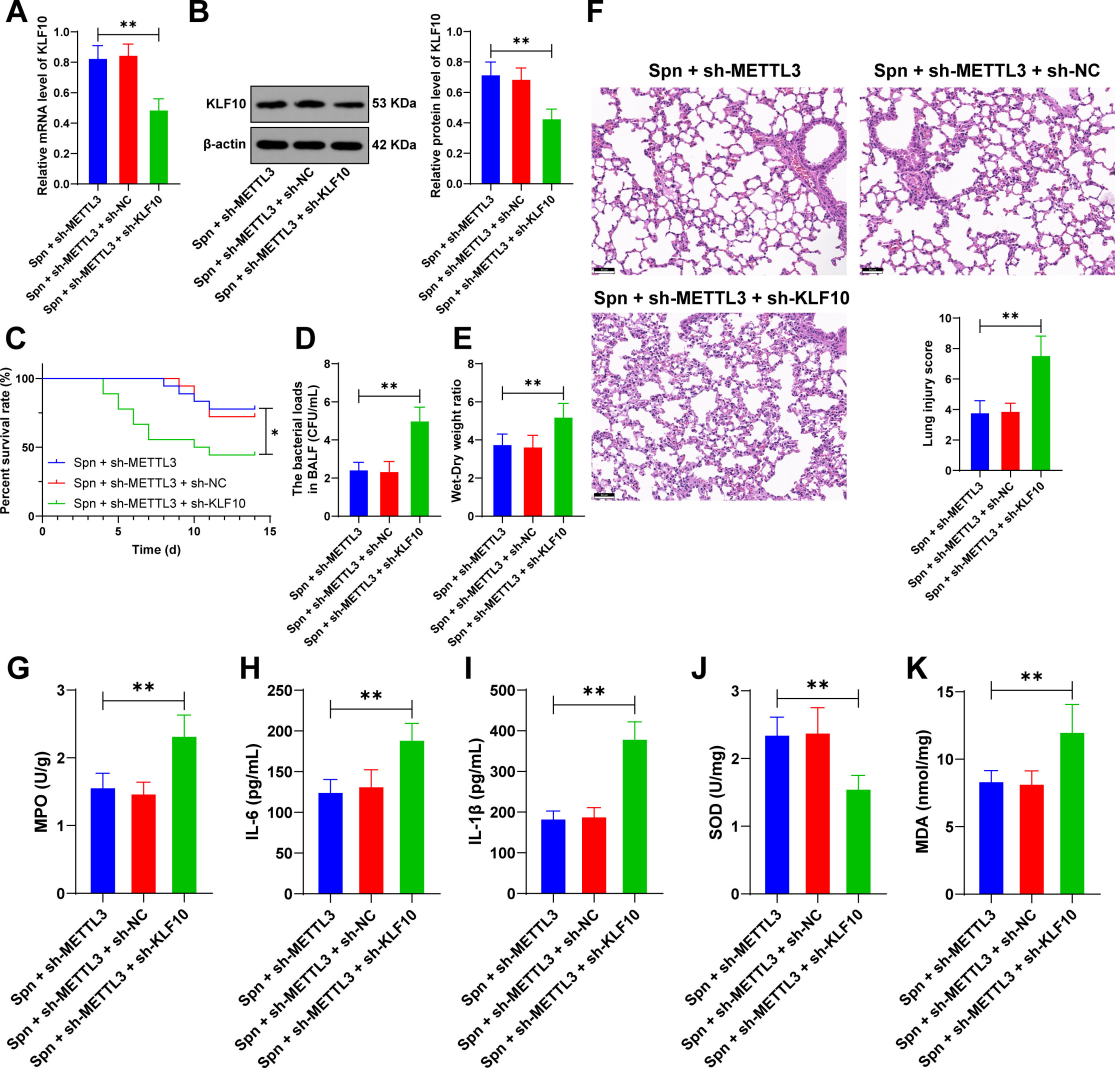
KLF10 downregulation alleviates the protective effect of METTL3 knockdown in neonatal mice with *Spn*-induced pneumonia. Lentivirus containing KLF10 shRNA (sh-KLF10) was injected via the tail vein into neonatal mice, with the sh-NC lentivirus-injected group serving as the negative control. Subsequently, the *Spn*-induced pneumonia model was established. (**A, B**) KLF10 expression in lung tissues was determined by RT-qPCR and western blot; (**C**) survival rates were recorded within 14 days; (**D**) bacterial load in BALF; (**E**) wet-to-dry weight ratio in the lung tissue; (**F**) H&E staining and a score system for evaluating the lung tissue damage; (**G**) MPO activity in the lung tissue; (**H**) IL-6 levels in the lung tissue were measured by ELISA; (**I**) IL-1β levels in lung tissue were measured by ELISA; and (**J**) SOD content was assessed using an assay kit; (**K**) MDA content was assessed using an assay kit. All animal experiments were independently repeated six times, with data presented as mean ± SD. Comparisons among multiple groups in (**C**) were analyzed by log-rank test and comparisons among multiple groups in (**A, B**) and (**D–K**) were analyzed by one-way ANOVA, followed by Tukey’s multiple comparisons test. * *P* < 0.05. ** *P* < 0.01.

Finally, to validate mechanistic findings, we reduced KLF10 expression (*P* < 0.01, Fig. 5A and B) in pulmonary tissue through lentiviral injection. KLF10 knockdown resulted in decreased survival rates and exacerbated lung injury severity in neonatal mice (*P* < 0.01, Fig. 5C through G). Additionally, KLF10 downregulation increased levels of IL-6, IL-1β, and MDA and suppressed the SOD activity (*P* < 0.01, Fig. 5H through K). These findings collectively demonstrate that KLF10 downregulation attenuates the protective effects of METTL3 knockdown against *Spn*-induced pneumonia in neonatal mice.

## DISCUSSION

Over the past few years, significant progress has been made in both clinical and basic research on the treatment strategies for lung injury. For infectious lung injuries, such as *Spn* pneumonia, antibiotic-based therapies remain the cornerstone (4, 28). However, current therapies face critical limitations: antibiotic resistance is escalating, and the metabolic differences of patients significantly affect efficacy (29). Against this backdrop, our study investigates the regulatory role of METTL3 in *Spn*-induced lung injury in neonatal mice, aiming to elucidate the molecular mechanism by which METTL3 modulates inflammatory factors via m6A modifications. Our results confirm that the bactericidal effect of the METTL3/KLF10 axis on *Spn* includes a direct effect on the inflammatory response and an indirect effect caused by the reduction of CFU. This exploration may pave a new way for novel therapeutic strategies targeting lung injury repair.

First, our experiment demonstrates that METTL3 is upregulated in lung injury of neonatal mice with *Spn*-induced pneumonia, and reducing METTL3 expression alleviates pneumonia. Indeed, METTL3-mediated m6A modification is critical for exacerbating lung injury. METTL3 exacerbates LPS-induced pulmonary inflammation by mediating m6A methylation of STAT2 mRNA to enhance its stability and translation, thereby activating inflammatory responses and aggravating lung injury (10). METTL3 aggravates pulmonary inflammation in bronchopulmonary dysplasia by mediating the m6A modification of ATG8, suppressing its expression, disrupting the GSDMD interaction, inhibiting autophagy, and inducing pyroptosis (30). Of note, following METTL3 downregulation, *Spn*-induced IL-10, IL-6, and TNF-α expression in alveolar epithelial cells was suppressed, leading to alleviated inflammation and reduced apoptosis (12). In our study, METTL3 upregulation exacerbated lung injury and reduced survival rate by increasing bacterial load in BALF, elevating lung wet-to-dry ratios and MPO activity, raising levels of IL-6, IL-1β, and MDA, and suppressing the SOD activity. In rats with *Spn*-induced pneumonia, a reduction in the bacterial load in BALF supports alleviated inflammation and improved prognosis (21, 31). Consistently, METTL3 inhibition reduced the bacterial load in BALF and alleviated lung injury, which provided a direction for the treatment of lung injury.

Furthermore, METTL3-mediated m6A modification plays a critical role in RNA stability, translational efficiency, and circRNA functionality (16, 32). For instance, m6A-modified circSAV1 promotes IREB2 mRNA translation, triggers ferroptosis in lung epithelial cells, and drives the progression of chronic obstructive pulmonary disease (33). Our experiment demonstrated that METTL3 could upregulate circ_0001239 expression via m6A modification, forming a novel regulatory axis in the pathogenesis of *Spn*-induced lung injury. Several circRNAs have been documented to worsen pneumonia and lung injury. In the LPS-induced 16HBE cell injury, upregulation of circ_0038467 promotes cell apoptosis and inflammation by sponging miR-545-3p (34). Similarly, circ_0012535 exacerbates pneumonia progression in fetal lung fibroblasts by targeting the miR-338-3p/IL6R signaling pathway to promote cell apoptosis and inflammatory responses (15). A recent report has revealed that elevated expression levels of circ_0001239 were observed in patients with community-acquired pneumonia (35). Here, circ_0001239 overexpression attenuated the protective effects of METTL3 knockdown on *Spn*-infected neonatal pneumonia in mouse models. Therefore, the METTL3-mediated m6A modification in circ_0001239 suggests a potential therapeutic strategy for pneumonia treatment.

Previous studies have shown that circRNAs can modulate RNA-binding proteins, thereby influencing downstream gene expression (36, 37). Circ_1304 increases YTHDF2 protein levels, leading to activation of VSMC autophagy (38). Similarly, our findings demonstrate that circ_0001239 interacts with YTHDC2, thereby disrupting KLF10 mRNA stability and exacerbating pneumonia-associated lung injury. The role of the KLF family in diseases and physiological events has been increasingly recognized (39). KLF9 aggravates inflammatory responses and lung injury by directly regulating gasdermin D expression (40). For an *in vitro* model of asthma, KLF12 overexpression suppresses LPS-induced inflammatory response and oxidative stress (41). KLF10 attenuates pulmonary inflammation by suppressing NPRA expression, playing a critical role in chronic lung disease pathogenesis (20). Our experiment aligns with this observation, demonstrating that circ_0001239/YTHDC2-mediated KLF10 upregulation alleviates *Spn*-induced lung injury, whereas KLF10 downregulation diminishes the protective effect of METTL3 knockdown in neonatal mice with *Spn*-induced pneumonia.

Our research still has several limitations. First, our investigation was limited to validating the downstream METTL3-mediated molecular mechanisms in a neonatal mouse model of *Spn*-induced pneumonia. The upstream regulatory mechanisms governing METTL3 expression and activity, however, have not been systematically investigated yet. Second, the specific m6A reader proteins involved in METTL3-mediated regulation of circ_0001239 have not been identified. Third, the ceRNA mechanism of circ_0001239 requires further verification. Fourth, although KLF10 was identified as a key transcription factor, its downstream signaling pathways remain unclear. Finally, our investigation was limited to inflammatory and oxidative stress factors, while the potential effects of METTL3 on pyroptosis and ferroptosis in the pulmonary tissues of pneumonic neonatal mice were not examined. Moving forward, the effect of METTL3 on pyroptosis and bacterial load in BALF will be further verified in animal experiments. Additionally, the upstream regulatory mechanisms of METTL3 and downstream regulatory mechanisms of KLF10 should be further investigated, which may lead to the identification of new therapeutic approaches for pneumonia intervention.

To summarize, our study unveils that METTL3 upregulates circ_0001239 expression via m6A modification, increases the binding of circ_0001239 to YTHDC2, and inhibits KLF10 expression, ultimately worsening lung injury in neonatal mice with *Spn*-induced pneumonia. Our studies provide a mechanistic framework for combining the m6A modifier with therapies targeting *Spn*-induced pneumonia, offering a promising way to alleviate lung injury.
